# Endoscopic Sleeve Gastroplasty for the Treatment of Obesity: A Single-Centre Experience and Literature Review

**DOI:** 10.7759/cureus.71064

**Published:** 2024-10-08

**Authors:** Agostino Fernicola, Giuseppe Palomba, Armando Calogero, Michele Santangelo, Alessandro Salvucci, Viviana Verlingieri, Giuseppe Scognamiglio

**Affiliations:** 1 Department of Clinical Medicine and Surgery, Division of Endoscopic Surgery, Azienda Ospedaliera Universitaria Federico II, Naples, ITA; 2 Department of Advanced Biomedical Sciences, Division of Emergency Surgery, Azienda Ospedaliera Universitaria Federico II, Naples, ITA; 3 Department of General Surgery, Ospedale Santa Maria della Pietà dei Religiosi Camilliani, Naples, ITA

**Keywords:** bariatric endoscopy, bariatric surgery, dysmetabolism, endoscopic procedures, endoscopic sleeve gastrectomy, endoscopic sleeve gastroplasty, obesity, overstitch system, surgical endoscopy, weight loss

## Abstract

Background: In the world, obesity is constantly increasing, and so are the types of medical and surgical therapies. However, to cope with the increase in costs associated with surgical procedures and certain complications, such as gastroesophageal reflux disease, the number of bariatric endoscopic (BE) procedures has been increasing in recent years. Endoscopic sleeve gastroplasty (ESG) is one of the most rapidly increasing BE procedures, given its benefits in terms of patient quality of life and reduced costs. In fact, it is a procedure characterised by minimal postoperative complications and is applicable to different types of bariatric patients. However, the number of studies on BE is not comparable to that on bariatric surgery.

Methods: We analyzed the results of 84 ESG performed in our centre and compared them with those presented in the literature. We evaluated 36-Item Short Form Health Survey (SF-36) to 0, six and 13 months, signs and symptoms of patients undergoing ESG on the first and second postoperative day (POD), in terms of abdominal pain, nausea and vomiting. We found a reduction of these outcomes from POD 1 to POD 2. Finally, we measured total body weight loss percentage (TBWL%) and excess weight loss percentage (EWL%) at three, six and 12 months both in patients undergoing bariatric surgery for the first time (specifically, ESG) and in patients previously undergoing bariatric surgery and then undergoing ESG.

Results: Using the SF-36 score, we observed an improvement in the physical (mean score from 46.4 at time 0 to 53.6 at 12 months from the ESG) and mental (mean score from 37 at time 0 to 39.9 at 12 months from the ESG) status of the patients. Furthermore, we observed a 0 to 12-month increase in TBWL% and EWL% from the ESG procedure. Furthermore, we observed an increase in TBWL% and EWL% at three, six and 12 months both in patients undergoing bariatric surgery for the first time (specifically ESG) and in patients previously undergoing bariatric surgery and then undergoing ESG.

Conclusion: ESG was an effective, reversible, and repeatable surgical procedure for bariatric patients.

## Introduction

The increasing incidence of obesity worldwide is associated with increased morbidity and mortality [1,2]. This has many economic implications in terms of healthcare costs, productivity, disability, and the potentially increased need for caregivers [2]. In response to this global epidemic, the rates of bariatric surgery increased [1,3]. In particular, sleeve gastrectomy (SG) is currently the most frequently performed bariatric surgical procedure in the world [4]. Although technically safe and effective for weight loss, SG may be associated with increased costs secondary to possible adverse events [3,4]. In fact, there may be an increased prevalence of de novo gastroesophageal reflux disease (GERD) or worsening of pre-existing GERD at surgery, with an associated increased long-term risk of Barrett's oesophagus or oesophageal adenocarcinoma [4]. In general, currently available interventions are effective but limited in their application by surgical indications, the patient's ability to accept the surgical procedure, and high intraoperative, postoperative and complication costs [3,4].

In this context, endoluminal techniques are emerging as effective therapies for the management of obesity [5]. Among the most widely used technologies, the intragastric balloon (IB) and the duodenal-jejunal bypass liner (DJBL) stand out [6]. Both techniques use the implantation of a device that remains in situ for no more than 12 months [2]. However, these therapies introduce the risk of device-related complications such as migration, gastrointestinal ulceration, and infection [7]. Furthermore, the implantation of the device is linked to a limited time, which increases the risk of regaining weight if lifestyle and eating behaviour changes are not adopted and maintained correctly [7].

From this perspective, endoscopic sleeve gastroplasty (ESG) is a safe and effective endoscopic therapy that does not require device implantation and represents one of the major advances in obesity therapy [5]. ESG is the endoscopic alternative to SG and gives satisfactory results in weight loss, although lower than those obtained with SG [8]. However, ESG has fewer adverse events and GERD, compared to SG [8]. The use of this endoscopic procedure has been steadily increasing over the years, and clinical studies reporting data obtained are necessary to clarify the validity of this bariatric approach [9]. However, scientific studies on this bariatric endoscopic (BE) procedure are not numerically comparable to those for bariatric surgical procedures [9]. For these reasons, the aim of this study was to report our experience with the ESG procedure and perform a review of the scientific literature.

The abstract of this work was presented at the XXXII Italian National Congress S.I.C.O.B. in May 2024.

## Materials and methods

Patient population and endpoints

We retrospectively studied all patients consecutively undergoing ESG at our hospital between March 1, 2021, and October 31, 2022, at the Santa Maria della Pietà Hospital of the Camillian Religious of Casoria, Naples, Italy. Clinical and demographic data included sex, age, body mass index (BMI), American Society of Anesthesiologists score (ASA score), clinical presentation, comorbidities, and history of previous bariatric surgery. We collected perioperative data from the computerized medical records in our hospital. The inclusion criteria were patients presenting a BMI between 30 and 35 kg/m^2^, patients not eligible for surgery due to high surgical risk, patients eligible for surgery who refused the surgery itself, or patients previously subjected to surgery (adjustable gastric banding, SG, mini bypass), in whom a recovery of more than 40% of the maximum weight lost from the surgery was observed. The exclusion criteria were patients with obesity presenting with severe gastritis or hiatal hernia larger than 5 cm or gastropathy due to portal hypertension, gastric polyps, or lesions of an undefined nature.

Preoperative examinations included psychological, nutritional, surgical, and anaesthesiologic evaluation, esophagogastroduodenoscopy (EGDS), and a quality-of-life assessment test (36-Item Short Form Health Survey, or SF-36). The SF-36 was performed at time 0 (preoperative visit) and consisted of 36 questions aimed at investigating the physical functioning and emotional well-being of the patient. We noted the symptoms reported by the patient and any complications and we performed regular follow-ups at three, six, and 12 months. During follow-up, patients completed the SF-36 and received a psychological and nutritional visit again and a reassessment of any postoperative complications.

The aim of this study was to evaluate the safety and efficacy of ESG in terms of physical and mental state reported at 0, six and 12 months using the SF-36 score and to define the total body weight loss percentage (TBWL%) and excess weight loss percentage (EWL%) at three, six and 12 months.

Materials and devices

In all endoscopic procedures, we have always used the following instruments. The endoscopic HeliX Tacking System (Apollo Endosurgery, Austin, TX) is a spiral instrument that we have inserted like a corkscrew into the gastric wall, allowing it to hook, lift, and bite the gastric wall at full thickness. We used the Overstitch System (Apollo Endosurgery) to perform a continuous 2/0 polypropylene suture starting from the anterior gastric wall at the angular notch, with subsequent bites performed on the greater curvature, towards the posterior gastric wall. The Overstitch System was mounted on a dual-channel gastroscope (Olympus Optica, Tokyo, Japan) to create multiple sutures. At the beginning and end of the endoscopic procedure, we always performed a control EGDS.

Surgical procedure

Preoperatively, all patients underwent an EGDS to rule out contraindications to the procedure, antibiotic prophylaxis, and prophylaxis for venous thrombosis. All patients provided written informed consent, before receiving the endoscopic bariatric surgery.

**Figure 1:**
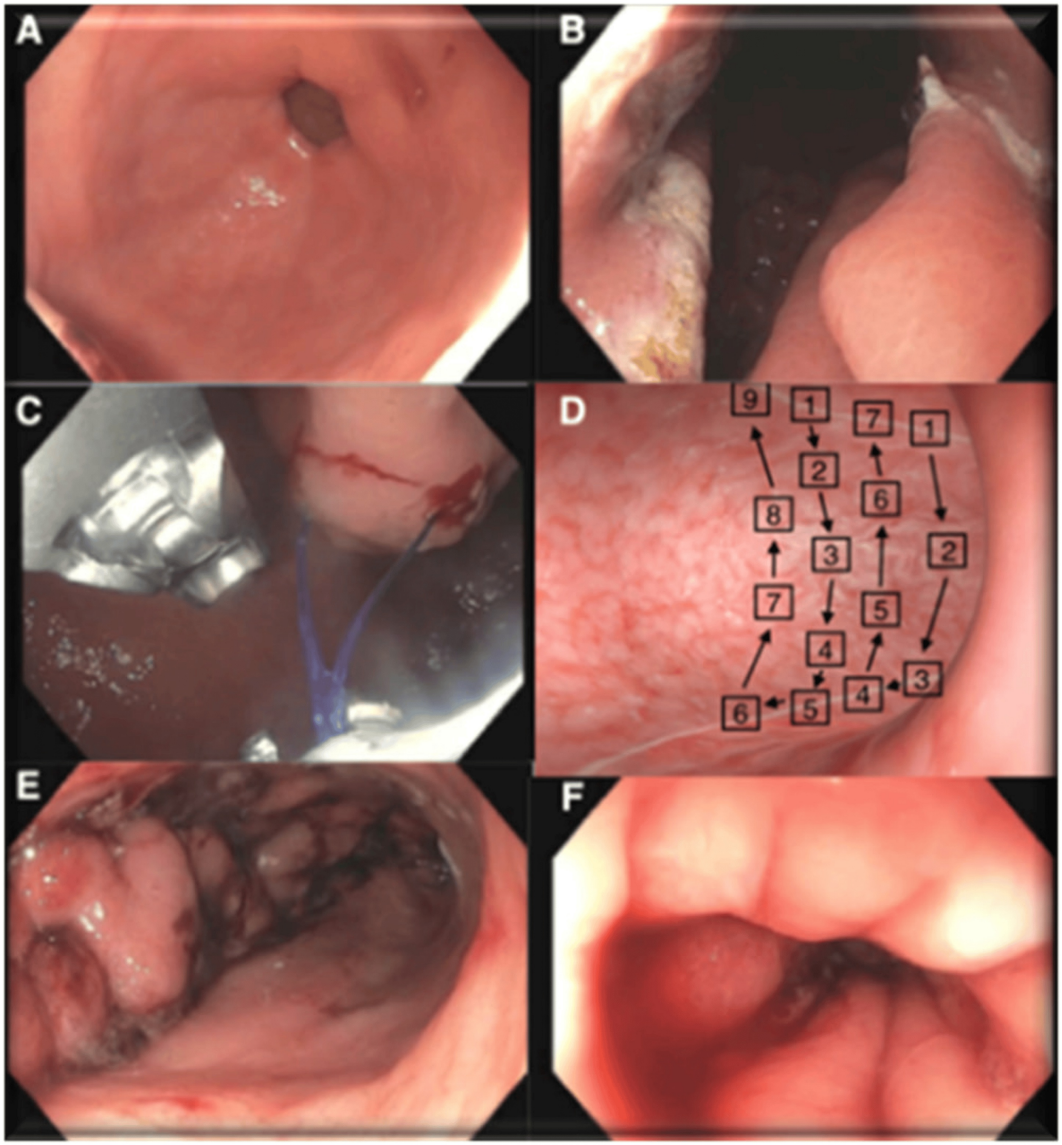
Endoscopic images during the ESG procedure (A) Visualization of the gastric antrum; (B) Demarcation of the plication sites; (C) Placement of the suture; (D) Stitch pattern used; (E) Result at the end of the procedure; (F) Result at three months. ESG: endoscopic sleeve gastroplasty

At the time of surgery, the patients were positioned in the supine position and underwent orotracheal intubation under general anaesthesia. The Overstitch System was attached to a double-channel gastroscope to facilitate the creation of multiple interrupted sutures. Each suture consisted of six to nine full-thickness bites of the wall of the greater gastric curvature to reduce the volume of the stomach from the angulus to the fundus, which was not touched. The gastric wall was hooked using the HeliX and a full-thickness Prolene 2/0 stitch (Ethicon, Inc., New Brunswick, USA) was placed endoscopically. The number of plications used was adjusted according to the length of the stomach, varying from three to six. The continuous suture began at the anterior wall of the stomach at the angular notch and continued, with bites on the greater curvature to the posterior gastric wall. We performed retrograde plication, 1 cm proximal to the initial suture, starting from the posterior wall towards the anterior wall, always at the greater gastric curvature. We placed the bites at full thickness of the proximal suture in a staggered manner compared with the distal suture. After completing the suture pattern, the needle was released, and the suture was tightened to fold the tissue. Finally, the thread was blocked and cut. We placed the sutures serially up, to 1 cm from the gastroesophageal junction, measured at the level of the lesser gastric curvature. Thus, at the end of the procedure, a small gastric fundus pouch remained. At the end of the procedure, we performed a new gastroscopy on all patients to ensure the absence of bleeding and optimal appearance of the sutures (Figure 1).

In the immediate postoperative period, the following were administered: omeprazole 20 mg, rociverine 10 mg, and metoclopramide as needed. On the first postoperative day, a transit X-ray was performed to evaluate the radiological image of gastric plication. If the result was normal, the patient began a semi-liquid diet. The morning after the procedure, patients underwent blood tests (complete blood count, creatinine, azotaemia, transaminasemia, bilirubinemia, glycaemia, amylasemia). Depending on these laboratories and radiological tests and in the absence of symptoms, we discharged the patients. All patients began a low-calorie liquid diet for at least three weeks, transitioning to a pureed and then solid diet within five weeks after the procedure. Each patient received a comprehensive complementary programme that included follow-up visits with endoscopists and allied health professionals (dieticians, behavioural psychologists, exercise physiologists). The programmes helped patients establish positive changes in diet and lifestyle.

Data collection and Institutional Review Board (IRB) details

The clinical and pathological data of 84 patients regarding the benefits and possible harms of this BE procedure were retrieved from the patient’s digital medical records and checked for accuracy. Data on age, sex, BMI, comorbidities, previous surgical interventions, average hospital stay, peri- and postoperative complications, and surgical duration operation time, the TBWL% and EWL% were extracted from the patient’s electronic records. By TBWL, we meant pre-op weight subtracted from post-op body weight. Weight was calculated in kilograms (kg). By TBWL%, we meant the fraction of body weight expressed in percentage terms. By EWL%, we meant the formula: (pre-op weight - follow-up weight)/(pre-op weight - ideal body weight) X 100. We did not perform a statistical analysis of our results because it was not necessary, as our data are in terms of percentage measurements and a scoring system for each patient that does not require p-values. The results of this study are reported in line with the Preferred Reporting of CasE Series in Surgery guidelines. We performed a review of the scientific literature by searching PubMed, Web of Science, Scopus and Embase for all studies on ESG, published from January 01, 1995, to August 31, 2024, using the following keywords: “Endoscopic Sleeve Gastroplasty” AND “ bariatric surgery” OR “bariatric surgery” OR “endoscopic procedures in bariatrics” OR “weight loss and bariatric endoscopy” OR “weight loss and Endoscopic Sleeve Gastroplasty” OR “dysmetabolism and Endoscopic Sleeve Gastroplasty”.

We waived IRB approval because this study retrospectively describes the results of an endoscopic technique (ESG) already approved and validated internationally by the scientific community and is not a clinical trial study; therefore, it was only necessary to have the patients sign informed consent.

## Results

Between March 2021 and October 2022, we collected data from 84 patients (48 patients were men and 36 were women) who underwent ESG, enrolled in a single centre consecutively. Of these, 44 patients underwent Redo-ESG (which we mean an endoscopic revision of a previous bariatric surgical procedure) of which 13 had previously undergone adjustable gastric banding (AGB), 13 mini gastric bypass (MGB), 15 SG, and three ESG. The mean BMI before treatment was 38 kg/m^2^, with a mode of 36 kg/m^2^. The mean duration of the procedure was 69 minutes (range 45-120 min). The number of sutures ranged between 3 and 5 (median = 4). Forty patients had never undergone previous bariatric surgery, and the ESG was their first bariatric procedure. Of the initial 84 patients, 83 were followed up to six months of follow-up, 81 were followed up to 12 months of follow-up, and only one patient never responded to the calls at six and 12 months of follow-up and was lost (Table 1).

**Table 1:** Short Form Health Survey 36 (SF-36) results

SF-36 Score	Time 0	6 Months	12 Months
Physical state	46.3	52.8	53.6
State of mind	37	44.9	39.9

Eighty-six per cent of the total patients reported experiencing post-procedural abdominal cramps or pain within 24 hours, along with 57% reporting transient nausea and 34% experiencing retching. On the second postoperative day, 33% of patients reported abdominal cramps/pain, 22% reported transient nausea, and 9% reported retching (Table 2). We encountered a case of bleeding that required endoscopic revision of the procedure with removal of the stitch at the site of the bleeding and direct mucosal hemostasis (Hb from 13.2 g/dL to 9.1 g/dL). The stitch was not replaced. We did not record any other adverse events. The mean hospital stay was two days. In the case of the bleeding described above, the hospital stay was four days. Of the 84 initial patients, we were able to collect data at six months for 83 patients and at 12 months for 81 patients.

**Table 2:** Percentage of patients with signs and symptoms on the first and second postoperative days 1 POD: first postoperative day; 2 POD: second postoperative day; n: absolute frequencies

Signs and Symptoms	1 POD (% of Patients)	2 POD (% of Patients)
Abdominal Pain	86% (n = 72)	33% (n = 28)
Nausea	57% (n = 60)	22% (n = 18)
Retching	34% (n = 29)	9% (n = 8)

After a 12-month follow-up, the TBWL% and the EWL% were on average 10.88% and 38.6%, respectively, with an increasing trend curve in the three, six and 12-month follow-up (Table 3). It is interesting to note how the results differ slightly when considering the two groups of patients: Redo-ESG and primary ESG. In the Redo-ESG group, mean values ​​of TBWL% were 7.19% at three months, 9.99% at six months and 11.21% at one year, and mean values ​​of EWL%were 23.4% at three months, 33.64% at six months and 31.1% at 12 months. In the Redo-ESG group, however, we observed TBWL% values ​​of 6.9% at three months, 8.4% at six months and 10.1% at 12 months as well as EBWL% values ​​of 27.2% at three months, 33.2% at six months and 39.1% at 12 months. The SF-36 score relating to physical health status improved from 46.3% at the beginning to 52.8% at six months, up to 53.6% at one year. The SF-36 mental status score increased from 37% at baseline to 44.9% at six months to 39.9% at 12 months.

**Table 3:** Three, six and 12-month follow-up in terms of TBWL% and EWL% TBWL%: total body weight loss percentage; EWL%: excess weight loss percentage; Redo-ESG: set of patients who have previously undergone another bariatric surgery procedure; Primary ESG: set of patients undergoing ESG for the first time; ESG: endoscopic sleeve gastroplasty

Redo-ESG	3 Months	6 Months	12 Months
TBWL%	7.19%	9.99%	11.21%
EWL%	24.3%	33.64%	31.1%
Primary ESG	3 Months	6 Months	12 Months
TBWL%	6.9%	8.44%	10.44%
EWL%	27.3%	33.2%	39.1%

## Discussion

The ESG is a transoral endoscopic procedure defined as incisionless in the international literature [5]. This condition causes a restriction of the gastric lumen at the body of the stomach through the application of a series of transmural sutures [5]. Using the endoscope-mounted suture platform Overstitch, continuous sutures are applied along the greater curvature of the stomach, resulting in a reduction in functional volume of approximately 70% [5,10]. In association with the plication of the greater curvature, the stomach is shortened by approximately 30% [9,10]. Current approaches spare the fundus, leaving an extremely small pouch [11]. ESG is the endoscopic alternative to SG and has a lower rate of postoperative complications than SG [8]. The weight results from ESG are satisfactory but lower than those of SG [8]. However, the reversibility, repeatability and safety of the procedure make ESG suitable for a greater number of obese patients, even as a bridge to SG for weight loss [8,12]. However, in the literature, the number of scientific works is not comparable to that of publications on bariatric surgical interventions. This study aimed to enrich the findings in the literature on ESG by analyzing the results of a single-centre experience on 84 consecutively treated patients.

The Mayo Clinic was the first to report the feasibility of the procedure in 2013 [9]. Two other centres subsequently adopted the procedure in the early stages of its development (Weill Cornell Medicine, New York, USA and Madrid Sanchinarro University Hospital, Madrid, Spain). The three centres combined the results of their data on 242 patients demonstrating a TBWL% of 15.2% at six months after the procedure and a TBWL% of 18.6% at 24 months after the procedure [9].

One of the most significant studies in this regard was the MERIT study: a randomised clinical trial conducted in nine US centres, aimed at evaluating the efficacy and safety of ESG [13]. In this trial, patients aged 21 to 65 years with class 1 or 2 obesity were enrolled, randomly assigned to the ESG group (ESG + lifestyle modifications) or to the control group (lifestyle modifications only), with potential confirmation or crossover at 52 weeks of ESG [13]. Lifestyle modifications included a hypocaloric diet and physical activity [13]. Participants in the ESG group were followed up for 104 weeks. The primary endpoint at 52 weeks was EWL% [13]. The secondary endpoint was the change in metabolic comorbidities between the two groups [13]. Between December 20, 2017, and June 14, 2019, 209 participants were assigned to the ESG group (n = 85) or the control group (n = 124). At 52 weeks, the mean EWL% was 49.2% for the ESG group and 3.2% for the control group (p<0.0001). The mean TBWL% was 13.6% for the ESG group and 0.8% for the control group (p<0.0001). Additionally, 59 of 77 participants in the ESG group (77%) achieved 25% or greater EWL at 52 weeks compared with 13 of 110 participants (12%) in the control group (p<0.0001). At 52 weeks, 41 of 51 participants (80%) in the ESG group had an improvement in one or more metabolic comorbidities versus 28 of 62 participants (45%) in the control group. At 104 weeks, 41 of 60 participants (68%) in the ESG group maintained 25% or more of their EWL. There were three ESG-related complications that did not require intensive care or surgery. It can therefore be concluded that ESG is a safe procedure that results in significant weight loss maintained at 104 weeks with important improvements in metabolic comorbidities and that this procedure should be considered as an adjunct tool for patients with class 1 or class 2 obesity [13]. The effectiveness of bariatric procedures is measured in terms of weight loss and metabolic effects. Among the available studies, that of Alqahtani et al. is one of the few studies to describe the metabolic benefits that follow ESG [14]. The authors reported a complete remission of diabetes in 76.5% of patients three months after the procedure [14]. Furthermore, all cases of hypertension (28 patients) and 56.3% of cases of dyslipidaemia went into remission within 12 months of follow-up [14]. The metabolic benefits of ESG have also been demonstrated by Sharaiha et al. [11]. The authors demonstrated a significant improvement in glycated haemoglobin at 12 months after the procedure, with a reduction in systolic blood pressure (from 129 ± 13.44 mmHg to 122.2 ± 11.69 mmHg, p = 0.023) and serum triglyceride levels (from 131.84 ± 83.19 mmol/dL to 92.36 ± 39.43 mmol/dL, p = 0.017). A reduction in serum alanine aminotransferase (ALT) values ​​at 12 months was also observed (from 42.4 U/L to 22 U/L in men and from 28 U/L to 20 U/L in women). Furthermore, the technical advantages of ESG are reversibility and repeatability. ESG is reversible because the stitches can be removed endoscopically and safely. Finally, this endoscopic procedure is repeatable not only for patients who have previously undergone ESG but also for those who have previously undergone bariatric surgery such as mini bypass or SG [11].

A recent study using a subset of the population of Sharaiha et al. analyzed leptin and insulin-glucose metabolism in post-ESG patients [15]. A total of 203 patients who underwent ESG were up to measure leptin and insulin levels. There was a significant decrease in insulin resistance and leptin (from 18.8 to 12.7 nm/mL, p = 0.05) at six months after the procedure.

In the meta-analysis conducted by Hedjoudje et al., all peri-procedural adverse events were reported [16]. The rate of severe post-ESG adverse events is considered to be 2.2%, with moderate heterogeneity [16]. No patient died from the procedure [16]. Severe adverse events include pain or nausea requiring hospitalisation (1.08%), upper gastrointestinal bleeding (0.56%), perigastric leak or abdominal collection (0.48%), pulmonary thromboembolism (0.06%), and pneumoperitoneum (0.06%) [16].

In the same study cited above, Alqahtani et al. also described the rates of revision and conversion for the ESG procedure [14]. Of the 1000 procedures considered, 13 patients (1.3% of the total) underwent endoscopic revision of the procedure or conversion to surgery [14]. Specifically, five (0.5%) patients had a redo-ESG, and eight (0.8%) had a conversion to SG, where the primary indication for conversion was an unsatisfactory weight loss (TBWL% <5%) at six months after the procedure [14]. Redo-ESG is a plausible procedure in which the stomach returns to its original anatomy despite the previous procedure; any residual stitches can be removed endoscopically to make a new suture, or pre-existing stitches can be tightened [17]. The reversibility and ease of conversion of ESG to other bariatric procedures is important, especially given the increased performance of endoscopic procedures. Lopez-Nava et al. found that conversion is reasonably simple given the preservation of gastric tissue after ESG [9]. In another study, Alqahtani et al. reported technical details and an evaluation of procedural aspects of converting an ESG to SG in 20 patients [18]. Specifically, an endoscopic and laparoscopic procedure was combined with initial endoscopic removal of the sutures. The authors reported no differences in complications or short-term weight loss compared with primary SG. Data on the conversion of ESG to Roux-en-Y gastric bypass are not yet sufficient, with the exception of case reports that underline the feasibility of the intervention provided that the sutures that could cause problems when using the stapler are removed [16].

Furthermore, Khan et al. compared ESG with POSE (primary obesity surgery endoluminal) in a systematic review, observing that the mean weight loss in terms of EWL% between the two procedures was 6.17 at six months and 7.84 at 12 months, in favour of ESG [19]. Similar results were obtained in the study published by Gys et al. who examined eight clinical trials with a focus on ESG: the EWL% at six and 12 months was significantly higher for patients who underwent ESG compared with POSE [20].

The results of our study confirmed that ESG is a safe and effective weight loss procedure with an excellent cost-effectiveness ratio. Quality of life was significantly improved. Stomach cramps and nausea were the most frequent adverse events, and they were easily manageable and resolved within a few days. Recovery was extremely rapid, allowing an immediate return to normal daily activities. Our result in terms of TBWL% at one year is lower than the result reported by a large prospective observational study conducted on 1000 consecutive patients with a mean BMI of 33.3 kg/m^2^ (10.88% vs 14.8%) [14]. However, it is adequate compared with what is proposed by the American Society for Gastrointestinal Endoscopy - Preservation and Incorporation of Valuable Endoscopic Innovations (ASGE-PIVI) guidelines (EWL% >25% and TBWL% >5%, maintained at one year) [21]. Patients with TBWL% >15 and EWL% >35 were considered an excellent result in light of the data reported in the literature, a result achieved in approximately 30% of patients [12].

Bariatric endoscopy can have beneficial metabolic effects and can fill the gap between medical and surgical therapies in a cost-effective manner. Being a low-risk and incisionless treatment, it significantly expands the catchment area, offering a valid treatment option to obese patients who refuse surgery, who are not considered fit for the surgery itself, or who have a BMI >30 kg/m^2^ but <35 kg/m^2^ without comorbidities. The possibility of proposing the procedure to individuals with various degrees of obesity who cannot undergo surgery is therefore confirmed. However, randomized controlled trials (RCTs) should be performed to evaluate long-term functional outcomes.

The limitations of our study are the relatively small number of patients enrolled and the follow-up of 12 months, and this is a single-centre study. Furthermore, another limitation is the lack of a statistical analysis that we chose not to conduct given the short follow-up and the absence of a control group. Obviously, the lack of statistical analysis and the presence of a control group makes it less easy to assess the full impact of ESG. Furthermore, the potential for bias in self-reported quality-of-life assessments is another element to consider. However, the short follow-up of 12 months allowed us to almost eliminate the risk of loss over time of patients undergoing ESG. Thus, the results obtained are satisfactory and comparable with those present in scientific literature. Finally, a limitation of the study is its retrospective nature, which makes it impossible to select patients later. This prompted us to design the study with extreme care, describing the methodology in detail in order to allow the replicability of the study. The future plan is to replicate a multicenter study with a larger sample of patients and a follow-up of 24 months.

## Conclusions

In our experience, we can say that ESG provides excellent results in terms of length of hospital stay, rapid control of immediate postoperative symptoms (nausea and cramps), and rapid recovery of daily activities. The procedure is also reversible, repeatable and safe. ESG may represent a bridge therapeutic opportunity for obese patients who are unwilling or unable to undergo bariatric surgery. Finally, ESG is not a contraindication to abdominal surgery: abdominal reaction to the endoscopically positioned stitches does not hinder the surgical procedure, and their endoscopic removal, at the same time as or before the procedure, avoids any difficulties associated with the use of a stapler at the time of gastro-resection.
